# Off-treatment virologic relapse and outcomes of re-treatment in chronic hepatitis B patients who achieved complete viral suppression with oral nucleos(t)ide analogs

**DOI:** 10.1186/1471-2334-14-439

**Published:** 2014-08-13

**Authors:** Hyung Rae Sohn, Bo Young Min, Joon Chang Song, Mun Hyuk Seong, Sang Soo Lee, Eun Sun Jang, Cheol Min Shin, Young Soo Park, Jin-Hyeok Hwang, Sook-Hyang Jeong, Nayoung Kim, Dong Ho Lee, Jin-Wook Kim

**Affiliations:** Department of Medicine, Seoul National University Bundang Hospital, 300 Gumi-dong, Bundang-gu, Seongnam-si, Gyeonggi-do 463-707 South Korea; Department of Internal Medicine, Seoul National University College of Medicine, Seoul, 110-799 South Korea

**Keywords:** Chronic hepatitis B, Discontinuation, Nucleos(t)ide analog, Relapse, Sustained response

## Abstract

**Background:**

The durability of off-treatment virologic responses has not been fully elucidated in chronic hepatitis B (CHB) patients who have previously achieved complete virologic suppression with nucleos(t)ide analog (NA) therapy. This study aimed to assess off-treatment virologic relapse rates and to characterize the outcomes of subsequent re-treatment in CHB patients who have discontinued oral NA following complete virologic suppression.

**Methods:**

Ninety-five CHB patients who showed complete virologic suppression were withdrawn from NAs: entecavir, lamivudine, and clevudine in 67, 15, and 13 patients, respectively. Consolidation therapy was given for 6 and 12 months for HBeAg-positive and -negative CHB, respectively, before cessation. Virologic relapse was managed with the same NA that had induced complete virologic response before discontinuation.

**Results:**

The cumulative rates of virologic relapse at 12 and 24 months were 73.8% and 87.1%, respectively. The relapse rates were independent of HBeAg positivity, HBeAg seroconversion, and type of oral NA. In a multivariate analysis, duration of oral NA therapy was the only significant predicting factor associated with off-treatment virologic relapse. Although the majority of patients regained complete virologic suppression, some patients did not respond to re-treatment with the initial NA and developed genotypic resistance.

**Conclusions:**

NA consolidation therapy for 6 and 12 months is associated with high off-treatment virologic relapse in HBeAg-positive and -negative CHB patients, respectively. Drugs with high genetic barriers to resistance should be considered as a rescue therapy for off-treatment relapse in CHB.

**Electronic supplementary material:**

The online version of this article (doi:10.1186/1471-2334-14-439) contains supplementary material, which is available to authorized users.

## Background

The goal of oral nucleos(t)ide analog (NA) therapy for chronic hepatitis B (CHB) is to suppress hepatitis B virus (HBV) replication in a sustained manner, preventing disease progression to decompensated cirrhosis and hepatocellular carcinoma (HCC) [1–5]. However, the duration of oral NA treatment required once complete virologic suppression is achieved has not been conclusively established. Although loss of HBsAg is the ideal endpoint associated with sustained off-treatment virologic suppression [6], HBsAg is cleared in a minority of CHB patients after antiviral therapy; only 5.6–11% of patients treated with pegylated interferon alpha clear HBsAg [7, 8], and the probability of clearance is even lower with oral NA therapy [6, 9]. HBeAg loss and/or seroconversion has been widely used as a surrogate endpoint of CHB therapy, and several practice guidelines suggest that oral NA treatment may be stopped after 6–12 months of consolidation therapy following HBeAg seroconversion [4, 5, 10, 11]. However, the study of off-treatment virologic response (VR) has been somewhat neglected. Although previous reports indicate that HBeAg seroconversion may not be durable [12–15], these data are from trials with lamivudine, a drug which is no longer recommended as initial therapy because of high resistance rates. Entecavir is widely used as a first-line therapy for CHB, but little is known about off-treatment VR. It is also yet to be elucidated whether re-treatment with the same oral NA can successfully re-induce complete VR.

The aims of this study were to assess off-treatment virologic relapse rates, identify predictive factors in CHB patients who achieved complete VR with NA therapy, particularly in those treated with entecavir, and to characterize the outcomes of antiviral therapy for off-treatment relapses.

## Methods

### Study design

Our study was a retrospective observational study carried out in a university-affiliated tertiary hospital in Seongnam, South Korea. Korean patients with CHB who had been on NA therapy and met the stopping criteria (described below) were eligible for discontinuation of oral NA therapy. Diagnosis of combined liver cirrhosis was made based on biopsy or a combination of clinical findings indicating the presence of portal hypertension. HCC was screened for and excluded throughout study period. The risk of viral reactivation was fully explained and discussed prior to cessation of medication. Patients who eventually stopped NA were identified through the hospital’s electronic medical record system (BESTCare) [16].

### Stopping criteria

The stopping criteria were as follows: for HBeAg-positive patients, at least 6 months of consolidation therapy after achieving HBeAg loss and complete VR (see below) [5, 13]; for HBeAg-negative patients, complete VR maintained for at least 12 months by consolidation therapy [11]. Exclusion criteria were: 1) total treatment duration <12 months; 2) decompensated cirrhosis; 3) patients who had received corticosteroid or anticancer chemotherapy; 4) patients who had comorbidities such as hepatitis C virus or human immunodeficiency virus infection, alcoholic liver disease, or autoimmune hepatitis.

### Definition of responses

After withdrawal of oral NA, conventional liver biochemistry, HBV serology, and serum HBV DNA levels were monitored every 1–3 months. Virologic relapse was defined as reappearance of serum HBV DNA >60 IU/mL, regardless of biochemical response. When virologic relapse was confirmed, a second course of treatment was considered with same oral NA that induced complete VR prior to discontinuation. The response to treatment was classified as follows: complete VR, a decrease in serum HBV DNA to undetectable levels, as determined by real-time quantitative polymerase chain reaction (PCR) assay (<60 IU/mL) or HBV DNA qualitative test; partial VR, a decrease in HBV DNA by >2 log_10_ IU/mL, but still detectable despite oral NA therapy for more than 6 months [17]; non-response, a decrease in serum HBV DNA by <2 log_10_ IU/mL after at least 6 months of therapy [5]. The Ethics Committee of the Seoul National University Bundang Hospital approved this study (IRB No. B-1205/156-113).

### Statistical analysis

Continuous variables were expressed as mean (±standard deviation) or median (range) where appropriate, and the Student’s t-test or Mann–Whitney U test were used with or without log transformation to compare two groups. Categorical variables were compared using the Chi-square test. Cumulative virologic relapse rates were estimated by Kaplan–Meier analysis, and the difference was tested by log-rank test. To identify independent predictors for off-treatment sustained VR, multivariate analysis was performed using binary logistic regression. Statistical analysis was performed with SPSS 17.0 (SPSS Inc.; Chicago, IL, USA). P-values of <0.05 were considered statistically significant.

## Results

### Off-treatment virologic relapse rate

**Figure 1:**
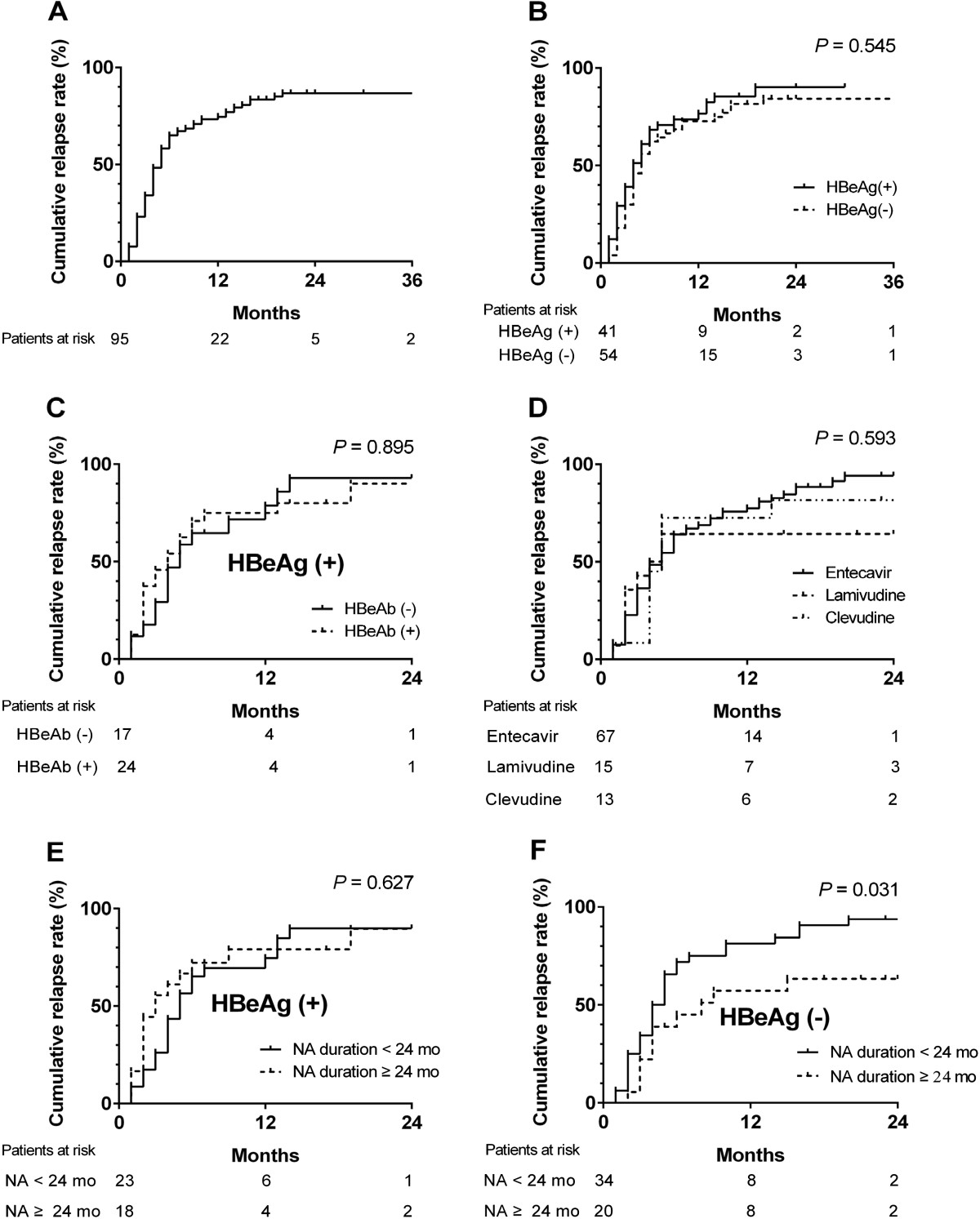
**Cumulative off-treatment virologic relapse rate.** The relapse rates at 12 and 24 months were 73.8% and 87.1%, respectively in patients with CHB who achieved complete viral suppression with oral nucleos(t)ide analog (NA) therapy **(A)**. The relapse rates were similar regardless of HBeAg status **(B)**, HBeAg seroconversion (for pre-treatment HBeAg-positive patients who subsequently lost HBeAg during treatment) **(C)**, and the type of oral NA: lamivudine 100 mg/day; clevudine 30 mg/day; entecavir 0.5 mg/day **(D)**. The relapse rates were not significantly different between HBeAg-positive patients with <24 months and >24 months of treatment **(E)**. However, the relapse rates were higher in HBeAg-negative patients who received >24 months of antiviral therapy compared with patients with shorter treatment duration **(F)**.

Between November 2004 and May 2010, NA treatment was prescribed to 2,736 patients with CHB. Of these, 236 patients stopped NA therapy for variable reasons between January 2006 and February 2012. Among these, 141 patients were excluded from further analysis because they did not meet the stopping criteria, rendering 95 CHB patients who discontinued oral NA after complete VR eligible for final analysis. The demographic, biochemical, and virologic profiles along with treatment course are summarized in Table 1. Of the 95 patients, 79 (83.2%) experienced virologic relapse and 16 (16.8%) patients remained in sustained remission during the median follow-up period of 22 months. Kaplan–Meier analysis showed that the relapse rates at 12 and 24 months were 73.8% and 87.1%, respectively (Figure 1A). Relapse rates were similar between HBeAg-positive and -negative patients (Figure 1B). In HBeAg-positive CHB, the relapse rate was not significantly different between patients with HBeAg seroconversion and patients with HBeAg loss only (Figure 1C). Different types of oral NA showed similar relapse rates (Figure 1D). Antiviral treatment for <24 months was associated with an increased virologic relapse rate compared with longer treatment duration in HBeAg-negative CHB patients (*p* = 0.031). In HBeAg-positive CHB, however, there was no statistically significant difference in relapse rates between treatment durations (Figure 1E, F).

**Table 1:** **Pre-treatment characteristics and treatment duration in CHB patients with or without off-treatment sustained virologic remission**

Factors	All patients	Sustained virologic remission	Relapse	P-value^a^
Number	95	16	79	
Age	47 (21–76)	45 (36–63)	49 (21–76)	0.242
Male (%)	53 (55.8)	9 (56.2)	44 (55.7)	0.968
Oral nucleos(t)ide				
Entecavir	67	8	59	
Lamivudine	15	5	10	0.110
Clevudine	13	3	10	
HBeAg (pos/neg)	41/54	6/10	35/44	0.616
HBeAb seroconversion^b^ before NA discontinuation	24	4	20	0.659
Alanine aminotransferase, xupper limit of normal	4.9 (0.4–30.8)	7.9 (1.1–30.8)	4.3 (0.4–25.4)	0.121
Prothrombin time, INR (range)	1.1 (1.0–1. 6)	1.1 (1.0–1.5)	1.1 (1.0–1.6)	0.704
HBV DNA (log IU/ml)	6.54 (3.31–8.00)	6.47 (3.31–8.00)	6.20 (3.44–8.00)	0.146
Cirrhosis	44 (46.4)	10 (62.5)	34 (45.0)	0.155
Total treatment duration (months)	22 (12–56)	28 (14–43)	22 (12–56)	0.016
Time to undetectable HBV DNA (months)	6 (1–23)	4 (2–6)	4 (1–23)	0.908
Additional treatment after HBeAg loss (months)^b^	14 (6–47)	16 (11–21)	13 (6–47)	0.988
Additional treatment after HBV DNA clearance^c^	16 (12–40)	25 (15–40)	17 (12–40)	0.001

Values are expressed as either median (range) or number (%).

^a^Sustained virologic remission vs. relapse.

^b^Data from HBeAg-positive CHB patients.

^c^Data from HBeAg-negative CHB patients.

### Predictors of off-treatment sustained virologic remission

Pre-treatment clinical and virologic characteristics were not significantly different between patients with or without sustained virologic remission (Table 1). However, total treatment duration and, in cases of HBeAg-negative CHB, additional treatment duration after HBV DNA clearance were significantly longer in the sustained virologic remission group. Multivariate analysis with a binary logistic regression model showed that treatment duration was an independent determinant of sustained virologic remission (odds ratio = 64.2, *p* = 0.042; Table 2).

**Table 2:** **Multivariate analysis for factors associated with off-treatment sustained virologic suppression**

Factors	Odds ratio (95% CI)	***P***-value
HBeAg positivity	1.43 (0.44–4.67)	0.554
Medication (lamivudine/clevudine vs. entecavir)	2.19 (0.66–7.20)	0.198
Total treatment (months)	64.20 (1.17–3530.37)	0.042

### Outcomes in patients with off-treatment virologic relapse

**Figure 2:**
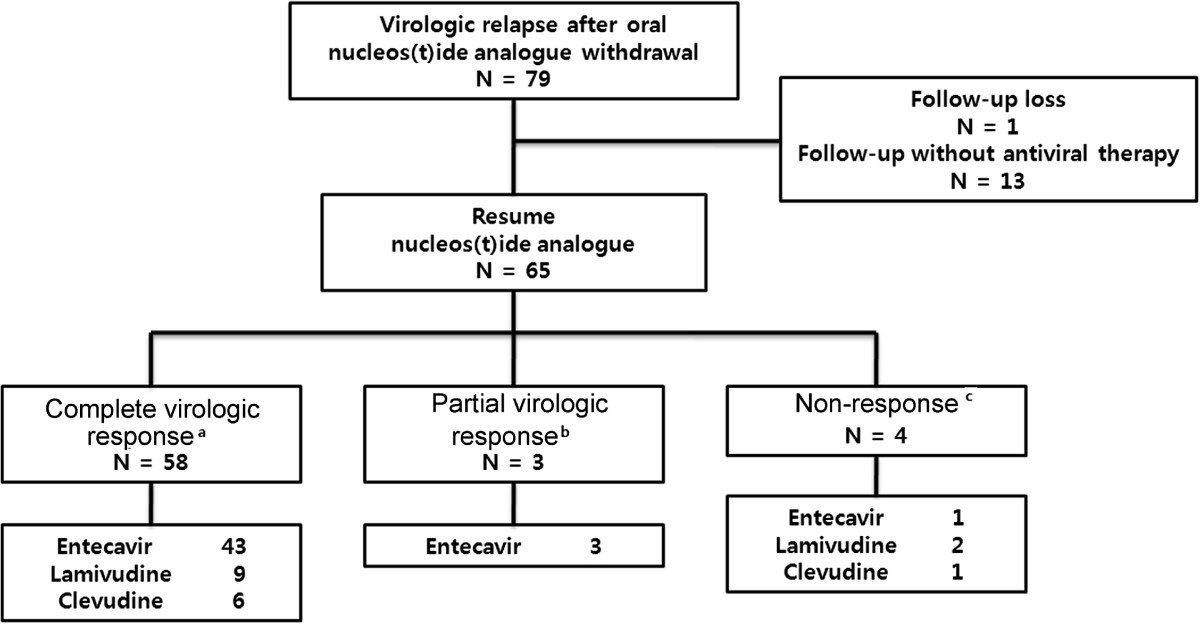
**Outcomes of CHB patients who experienced off-treatment virologic relapse after achieving complete viral suppression with oral NA.** Among the 65 patients who resumed the same oral NA that had induced initial complete virologic response, 58 patients again achieved complete VR, three patients achieved only partial VR, and four patients showed non-response. ^a^Undetectable HBV DNA titer; ^b^HBV DNA decreased by >2 log, but remained detectable; ^c^HBV DNA decreased by <2 log or increase.

Of 79 patients with off-treatment virologic relapse, one patient was lost to follow up and 13 patients were followed without further antiviral therapy. These 13 patients remained in biochemical remission and HBV DNA levels were maintained below 2,000 IU/mL. The remaining 65 patients received the same oral NA that had induced initial complete VR. Among them, 58 patients (89.2%) showed re-establishment of complete VR within 1–15 months (mean treatment time to HBV DNA loss was 3.5 months). However, three patients (4.6%) showed only partial VR and four patients (6.2%) showed non-response (Figure 2). Mutation analyses showed that resistance mutations were not observed in the complete or partial VR groups, whereas a lamivudine-resistant mutation (rtM204I/V) was found in all four non-responding patients. None of the relapsing patients experienced hepatic decompensation throughout the study period.

The clinical characteristics and outcomes of the four non-responding patients are summarized in Table 3. These patients had relatively high levels of HBV DNA and experienced early relapse within 6 months. After drug-resistant mutations were confirmed, patients who previously received clevudine/lamivudine therapy were given entecavir (1 mg), and patients with previous entecavir therapy received combination therapy of entecavir (1 mg) plus adefovir. Following these rescue therapies, two patients achieved complete VR, and two patients showed partial VR.

**Table 3:** **Characteristics of CHB patients who showed non-response to re-treatment with the same oral nucleos(t)ide analog that had induced initial complete virologic response**

Patients	Medication	HBeAg status	Baseline DNA level (IU/ml)	Liver cirrhosis	Total treatment^a^(months)	Additional treatment^b^(months)	Time to relapse^c^(months)	Genotypic resistance	Rescue therapy after second treatment failure	Response to rescue therapy
Patient 1	Clevudine	Positive	20,000,000	Negative	13	7	5	rtM204I	Entecavir	Sustained VR
Patient 2	Lamivudine	Negative	2,099,619	Positive	24	18	2	rtM204I	Entecavir	PartialVR
Patient 3	Lamivudine	Negative	257,039	Negative	20	7	3	rtM204I	Entecavir	Sustained VR
Patient 4	Entecavir	Positive	20,000,000	Negative	33	16	6	rtM204I	Entecavir + Adefovir	Partial VR

^a^Total duration of initial oral nucleos(t)ide analog therapy.

^b^Additional treatment duration of initial oral nucleos(t)ide analog therapy after undetectable HBV DNA.

^c^Time to relapse after discontinuation of initial oral nucleos(t)ide analog therapy.

VR, virologic response.

## Discussion

One of the unresolved issues in the management of CHB is how long oral NA must be maintained once patients have achieved complete virologic suppression. Current American Association for the Study of Liver Diseases (AASLD) guidelines recommend at least 6 months of additional treatment after HBeAg seroconversion [5]. However, about a quarter of patients experience relapse within 2 years of completing 6 months of lamivudine consolidation [12–15]; at 4 years, the relapse rate is increased to 82% after cessation of lamivudine [18]. In our study, about three quarters of CHB patients demonstrated off-treatment virologic relapse within 12 months of treatment termination. Although we used HBeAg loss rather than HBeAg seroconversion as a stopping criterion, subgroup analysis showed that the virologic relapse rates were not different between the seroconversion and HBeAg loss groups (Figure 1C; [13]). We believe that the high relapse rates in our study may be ascribed to different definitions of virologic relapse; previous studies used higher cut-off values of HBV DNA titer (10,000–100,000 copies/mL) whereas we used the 60 IU/mL (300 copies/mL) cut-off, determined by sensitive real-time PCR assays. Indeed, studies using similar cut-off values showed similar relapse rates in HBeAg-positive CHB [14, 19, 20]. These findings suggest that 6-month consolidation therapy is insufficient for preventing virologic relapse in HBeAg-positive CHB.

AASLD 2009 guidelines and European Association for the Study of the Liver (EASL) 2012 clinical practice guidelines do not recommend stopping NA treatment in HBeAg-negative CHB unless HBsAg is cleared [4, 5]. These recommendations are based on the observations that treatment duration of less than 1 year was universally associated with relapse [21, 22]. However, viral suppression may not have been complete at the time of treatment cessation in these studies as the cut-off for the definition of complete VR was relatively high (0.7 Meq/mL; 150,000 IU/mL). Indeed, more stringent stopping criteria with extended treatment duration resulted in up to 50% of remission rate in HBeAg-negative CHB [23, 24]. Asian-Pacific guidelines, conversely, state that treatment discontinuation can be considered after 12–24 months of consolidation therapy [11, 25]. We wanted to know the off-treatment remission rate after achieving complete viral suppression confirmed by a sensitive PCR method. Our data showed that 12-month consolidation therapy after serum HBV DNA loss was associated with high virologic relapse rates, similar to those of HBeAg-positive CHB with 6-month consolidation. Again, the high relapse rates in our study may be due to lower cut-off levels, determined by real-time quantitative PCR, compared with previous reports [23, 24].

Current guidelines recommend entecavir and tenoforvir as first-line drugs because of their high potency, high genetic barrier against resistance, and excellent long-term viral suppression [4, 5]. Patients in this study started oral antiviral therapy between 2004 and 2010; however, entecavir and tenofovir were not approved for the treatment of CHB in Korea until 2008 and 2013, respectively. Therefore, lamivudine was prescribed until 2008, after which entecavir was chosen as a first-line NA for the treatment of CHB, while tenoforvir was not available to these patients at any time. Although entecavir shows higher virologic suppression rates compared with lamivudine [26], off-treatment relapse was scarcely studied compared with lamivudine. Previous reports detailed relapse data after protocol-defined termination without consolidation therapy [19, 22, 26, 27]. We found that the off-treatment relapse rates were similar between entecavir and the less potent drug, lamivudine. Previous phase III clevudine trials showed delayed off-treatment virologic relapse [28, 29] but again, the off-treatment VR rate was similar. This result suggests that once complete virologic suppression is obtained, off-treatment remission rates are independent of the antiviral potency of the NA in question, and also suggests that the stopping criteria for entecavir should be as strict as those for less potent drugs. Because this study did not compare head-to-head off-treatment remission rates between entecavir and other NAs, further prospective studies with longer follow-up periods will be necessary to address this issue.

The univariate and multivariate logistic regression analyses showed that duration of NA therapy is an independent predictor of off-treatment sustained virologic remission in CHB. This finding is in line with previous studies of CHB patients treated with lamivudine [13–15]. Considering the high off-treatment relapse rates in our study, 6 months of consolidation is insufficient in HBeAg-positive CHB patients, as recommended by the recent EASL guidelines [4]. However, the Kaplan–Meier analysis in our study did not show a significant difference in relapse rate according to treatment duration (<24 mo vs. >24 mo) in HBeAg-positive CHB, and consolidation of more than 12 months did not decrease relapse rates compared with a shorter consolidation period (data not shown). The reason that extension of consolidation failed to reduce virologic relapse in HBeAg-positive CHB is not clear at present, but our finding suggests that even longer consolidation may be necessary in this group. Off-treatment response is more difficult to predict in HBeAg-negative CHB because no surrogate markers are available, except for the rare event of HBsAg loss. We found that prolonged consolidation is more effective for induction of sustained virologic remission in HBeAg-negative CHB compared with HBeAg-positive disease. Thus, we suggest that duration of oral nucleos(t)ide therapy be no less than 2 years in HBeAg-negative CHB. Further prospective randomized controlled studies are needed, however, to determine the optimal duration of NA consolidation in CHB.

Because more than 60% of patients still experience virologic relapse after more than 2 years of NA therapy in HBeAg-negative CHB, current guidelines for long-term antiviral maintenance until HBsAg loss may be a safe approach, especially for patients with liver cirrhosis [4, 5]. However, because some patients show sustained off-treatment virologic remission in our study (10/54, 19%) and other reports [23, 24], cessation of oral NA might also be an clinical option in HBeAg-negative CHB patients with or without liver cirrhosis who show complete VR for more than 2 years [25] if significant adverse events can be prevented in relapsers. We closely monitored relapsers and NAs were resumed before biochemical relapse if HBV DNA increased over 2,000 IU/mL. Although about half of our study patients had liver cirrhosis, no hepatic decompensation was documented after virologic relapse. A similar study regarding the off-therapy durability of entecavir published recently reported that only one patient, who did not conform to the follow-up schedule, developed decompensation, which was successfully managed by a second course of entecavir treatment [30]. Thus, cessation of NAs may safely be tried in patients with cirrhosis on the condition of close monitoring.

There is no consensus on how to manage off-treatment HBV relapse. Our results revealed that about 10% of patients with off-treatment relapse failed to regain complete virologic suppression with the same NA regimen. Interestingly, all four patients with non-response to the initial NA regimen developed the rtM204I mutation during the course of secondary treatment. Therefore, drugs with high genetic barrier should be considered as a rescue therapy for off-treatment relapse in CHB, and early evaluation for genotypic resistance should be considered in these patients if the initial regimen shows inadequate response.

A relatively small sample size is the major limitation of our study. However, considering the current trend, which does not recommend early termination of NAs, results from larger prospective trials may not be readily available. In the meantime, our data may help when making clinical decisions where long-term maintenance of NAs is not feasible.

## Conclusion

In summary, sensitive HBV DNA assays show high off-treatment VR rate in CHB patients who received consolidation therapy (6 and 12 months for HBeAg-positive and -negative CHB, respectively). Prolonged consolidation may reduce off-treatment relapse in HBeAg-negative CHB. Although most patients with virologic relapse regain complete virologic suppression with the same drug, some patients do not respond to re-treatment with the initial NA and develop genotypic resistance. Therefore, NA with a high genetic barrier should be considered as a rescue therapy for off-treatment relapse in CHB.

## Authors’ original submitted files for images

Below are the links to the authors’ original submitted files for images. Authors’ original file for figure 1 Authors’ original file for figure 2

## Abbreviations

NA: Nucleos(t)ide analog
CHB: Chronic hepatitis B
HBV: Hepatitis B virus
HCC: Hepatocellular carcinoma
HBsAg: Hepatitis B surface antigen
HBeAg: Hepatitis B e antigen
VR: Virologic response
AASLD: American Association for the Study of Liver Diseases
EASL: European Association for the Study of the Liver.
