# Supplementary figures and images for: Off-treatment virologic relapse and outcomes of re-treatment in chronic hepatitis B patients who achieved complete viral suppression with oral nucleos(t)ide analogs
Source: BMC Infect Dis. 2014 Aug 13;14:439. doi: 10.1186/1471-2334-14-439 (PMC4148928; doi:10.1186/1471-2334-14-439)

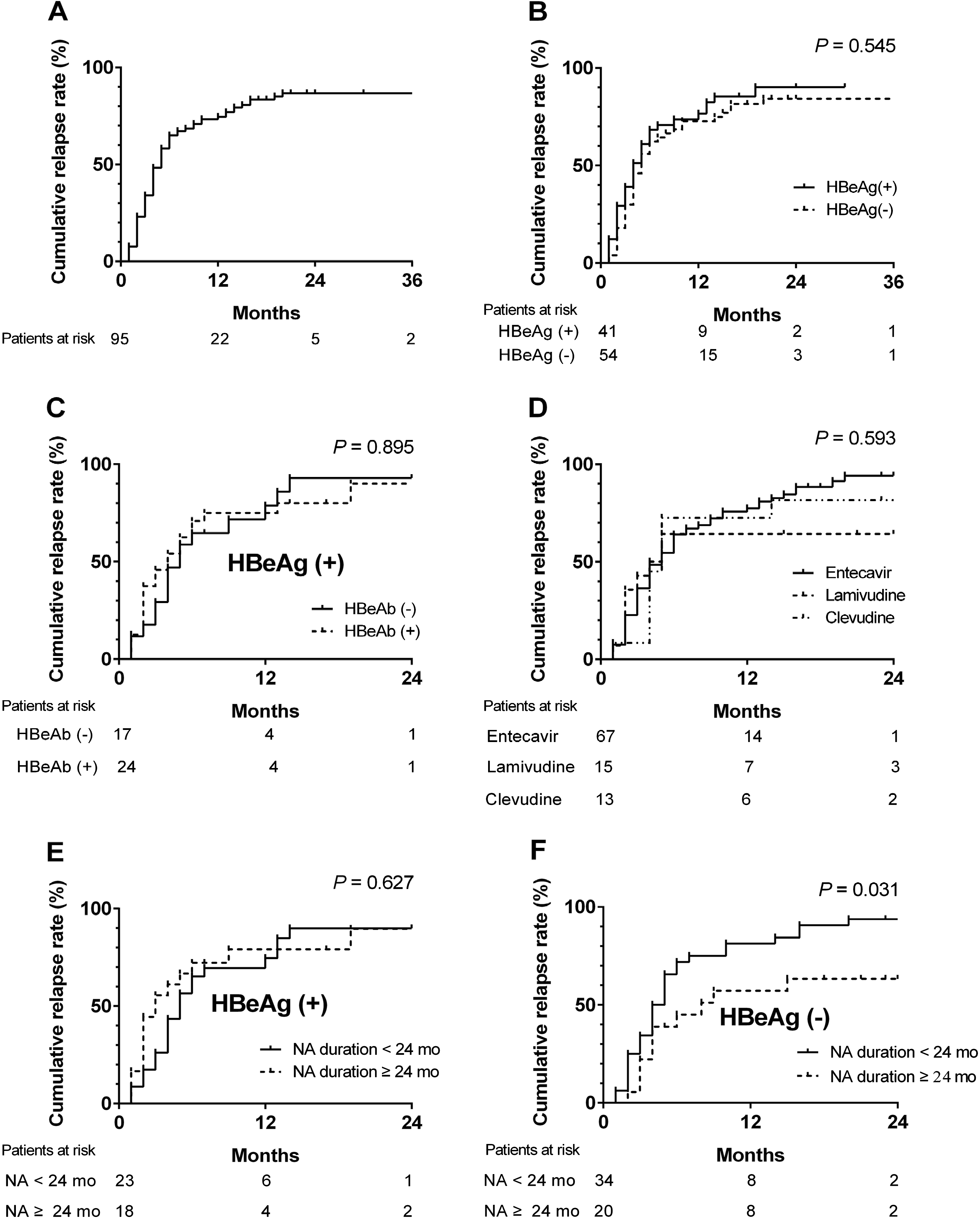

Supplement: Supplementary file 1 — Authors’ original file for figure 1 [file 12879_2013_3749_MOESM1_ESM.tif]

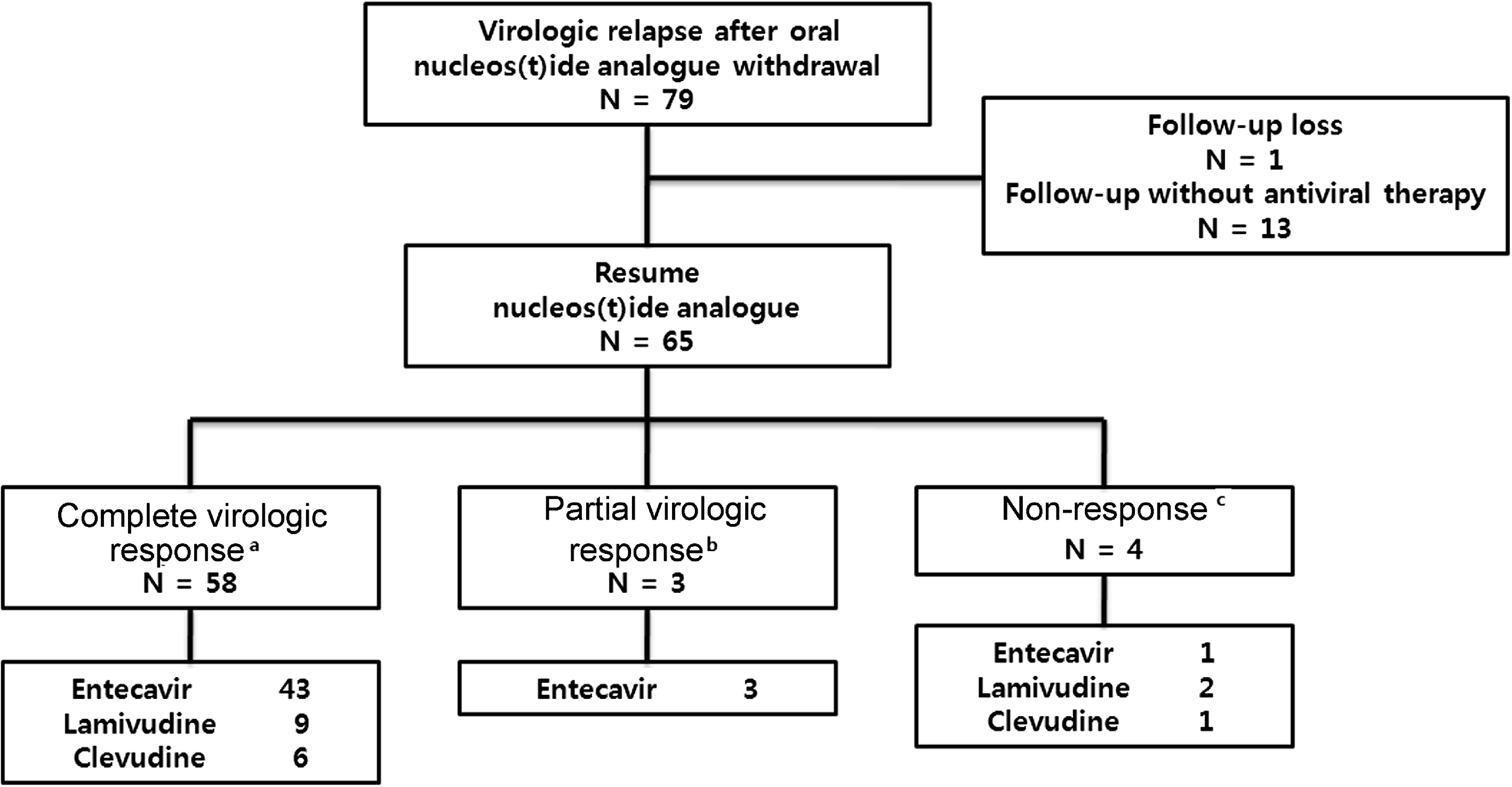

Supplement: Supplementary file 2 — Authors’ original file for figure 2 [file 12879_2013_3749_MOESM2_ESM.tif]
